# *Salmonella* Excludes *Salmonella* in Poultry: Confirming an Old Paradigm Using Conventional and Barcode-Tagging Approaches

**DOI:** 10.3389/fvets.2018.00101

**Published:** 2018-05-16

**Authors:** Yichao Yang, Guillermo Tellez, Juan D. Latorre, Pamela M. Ray, Xochitl Hernandez, Billy M. Hargis, Steven C. Ricke, Young Min Kwon

**Affiliations:** ^1^Department of Poultry Science, University of Arkansas, Fayetteville, AR , United States; ^2^Department of Veterinary Pathobiology, Texas A&M University, College Station, TX, United States; ^3^Facultad de Medicina Veterinaria y Zootecnia, Universidad Nacional Autónoma de México, Mexico City, Mexico; ^4^Cell and Molecular Biology Program, University of Arkansas, Fayetteville, AR, United States; ^5^Center of Food Safety, Department of Food Science, University of Arkansas, Fayetteville, AR, United States, 72704, USA

**Keywords:** *Salmonella*, poultry, competitive exclusion, barcode-tagged isogenic strain, intestinal colonization

## Abstract

*Salmonella* is one of the major foodborne bacterial pathogens, and the consumption of contaminated chicken meats isa primary route of *Salmonella* transmission into human food chains. However, the mechanism of *Salmonella* transmission within the chicken flock is not fully understood, including competition among *Salmonella* strains during chicken infection. The purpose of the present study was to evaluate the competitive exclusion (CE) between different or same *Salmonella* species consecutively challenged through the oral route. Two different approaches were used to evaluate the CE effect, including tracking *Salmonella* colonization by wild-type strains with difference in natural antibiotic resistance or DNA barcode-tagged isogenic strains. When day-of-hatch chicks were administered by wild-type *S.* Typhimurium (ST) on day 1, followed by infection on day 2 by *S.* Enteritidis (SE) or vice versa, most of the birds were colonized only by the first strains administered (82% by ST or 83% by SE). When similar experiments were performed using two different isogenic barcode-tagged SE strains, Illumina sequencing analysis of the barcode region showed that the first barcode-tagged strains administered were dominant strains, ranging from 92 to 99% of the *Salmonella* recovered from ceca. These results provide quantitative evidence supporting the CE theory that oral administration of *Salmonella* will produce predominant inhibition over the subsequent colonization of ceca by the following administration one day later by different or same *Salmonella* species. We also showed that the use of barcode-tagged isogenic strains in combination with deep profiling of barcodes by Illumina sequencing can serve as a quantitative method for studying complex dynamics of *Salmonella* infection, transmission and colonization in poultry.

## Introduction

Due to the common foodborne illness cases caused by *Salmonella*, prevention of *Salmonella* colonization in the gastrointestinal tract (GIT) of chickens is necessary. Because normal gut microbiota are not fully developed until 3–6 weeks of age, chicks are particularly vulnerable and susceptible to infection by *Salmonella* (1, 2). The live attenuated *Salmonella* vaccine strain has been identified as an effective approach for controlling gut colonization by pathogenic *Salmonella*. These vaccine strains are believed to reduce the ability of *Salmonella* to colonize in the chickens primarily via stimulating cell-mediated immunity (3, 4). However, another possibility is that vaccination may do so via modulating the diversity and structure of gut microbiome in the chicken (5). Crhanova et al. (6) also suggested that attenuated *Salmonella* vaccines are able to modify the chicken gut microbiota, enhance the maturity of gut immune system, and subsequently increase resistance to infection by pathogenic strains. However, these may not be the only mechanisms by which a decrease in *Salmonella* colonization occurs in the chickens. As compared to stimulation of the immune system and modulation of gut microbiota, which takes a longer time, competitive exclusion (CE) mechanism might be acting for immediate effect in suppressing other *Salmonella* strains (6). Methner et al. (7) proposed that vaccination of chicks at day one post-hatch ensures colonization by the live attenuated *Salmonella* vaccine strain, which produces an inhibitory effect and stimulates the development of an immunological response to the following infection (7).

Barrow et al. (8) also demonstrated that oral administration of live strains of virulent *Salmonella* to day-old chicks produced inhibition in the subsequent cecal colonization by *Salmonella* Typhimurium (ST) strain administered one day later. Interestingly, closely related enterobacteria were unable to induce the same effect (8). Rabsch et al. (9), used mathematical models that combined epidemiology and population biology to postulate a theory that *Salmonella* Enteritidis (SE) occupied the ecological niche vacated by eradication of *Salmonella* Gallinarum (SG) and *Salmonella* Pullorum (SP) from poultry (9). The theory suggests that SG was able to competitively exclude SE from poultry flocks in the early 20th century, and the elimination of SG and SP in poultry led to an epidemic increase of SE in poultry and human infections in the 1980’s. Protection against colonization by SE, but not ST, through immunization of chickens with SG was possible because both SG and SE possess the same immune-dominant O-antigen on their cell surfaces (10–12). Holt and Gast (13) reported that prior infection of hens with ST or *Salmonella* Muenchen (SM) reduced the infection by SE, which indicated that cross-serovar protection occurred among ST, SM and SE (13). Growth inhibition by different serovars may also occur in a serovar-specific manner through inhibitory metabolites. For example, in an *in vitro* experiment conducted by Calo et al. (14), decreased growth of ST occurred in spent media originating from *S.* Heidelberg growth cultures, but not from other serovars (14).

CE is a theory in ecology, which states that two closely related species that compete for the same resources cannot stably occupy the same ecological niche. This CE concept has been explored extensively as an effective strategy to control *Salmonella* in poultry since the landmark research by Nurmi and Rantala (15). To better describe CE phenomenon that may exist between different or same *Salmonella* serovars accurately, it is important to employ a quantitatively approach. For evaluation of CE theory, strains carrying different antibiotic markers have been used widely to differentiate two different strains from tissues or organs with mixed infections. However, these marker strains may not behave exactly in the same manner as the corresponding wild type strains due to the phenotypic changes caused by antibiotic markers (16). Hence, it is critical to use the isogenic strains phenotypically identical for studying CE to decrease the bias from different infection strains. We have constructed a set of isogenic SE barcode-tagged strains in which unique DNA barcodes were inserted in a functionally neutral locus in the genome of SE and the resulting strains can be used to quantitatively track the colonization by the respective strains by profiling the barcode-regions using Illumina sequencing method (16). The main advantage of using these barcode-tagged strains over previously used marker strains is that each strain can be tracked quantitatively within the entire population of barcode-tagged strains at high accuracy. This similar strategy has been used in several studies to allow quantitative profiling among multiple barcode-tagged strains as well as discrimination from the environmental bacteria or viruses without altering phenotypes or behaviors during infection, colonization and dissemination (16–20). The purpose of the present study was to evaluate the CE responses of *Salmonella* consecutively challenged in chickens using both conventional and barcode-tagging approaches.

## Materials and Methods

### Experiment 1: Recovery of *Salmonella* After Consecutive *Salmonella* Serovar Challenges in Day-Old Lenghorn Chicks (Trials 1 and 2)

***Salmonella***** cultures**. A highly invasive poultry isolate of SE was obtained from the USDA National Veterinary Services Laboratory (Ames, IA 50011). A spontaneous mutant that is resistance to Nalidixic acid (NA; Sigma, St. Louis, MO) was selected and used in this study. This strain was also found to be naturally sensitive to gentamicin (GM10) on antibiotic sensitivity discs. A spontaneous mutant of an invasive isolate of ST (ATCC13311) that is resistance to NA was also selected and used in this study. This ST was found to be resistant to GM10. Both strains of SE and ST were inherently resistance to Novobiocin (NO). In Experiment 1, we conveniently used the difference in the sensitivity to gentamicin between SE (GM10^S^) and ST (GM10^R^) to differentiate these two strains. For the present studies, 100 µL of SE or ST from a frozen stock was added to 10 mL of tryptic soy broth (TSB, Becton Dickinson, Sparks, MD) and incubated at 37°C for 8 h. This was followed by 3 passages every 8 h into fresh TSB. After incubation, bacterial cells were washed 3 times in sterile 0.9% saline by centrifugation (1,864 × g, 4°C, 15 min), and quantified with a spectrophotometer (Spectronic 20D+, Thermo Scientific, Waltham, MA) at 625 nm using an established standard curve. The cell suspensions were then diluted in sterile 0.9% saline as per required concentrations for the corresponding trials. Concentrations of SE or ST were also determined retrospectively by plating appropriate serial dilutions onto brilliant green agar (BGA, Sigma) supplemented with NO (25 µg/mL) and NA (20 µg/mL) for enumeration of actual CFU/mL used for the challenge studies.

**Experimental birds.** Naïve, day-of hatch, single comb white Leghorn male chicks obtained from a local hatchery were randomly placed in cages (n = 30 for each Control group or n = 60 for Treatment group for Trial 1; n = 20 for each Control group or n = 40 for Treatment group for Trial 2) within electrically heated starter batteries. The cages were located within a modern biological hazard isolation unit on the research farm of the College of Veterinary Medicine, Texas A & M University (College Station, TX). Chicks were provided *ad libitum* access to water and a balanced unmedicated corn-soybean diet meeting the nutrition requirements of poultry recommended by the NRC (1994). Adequate body temperature was maintained using heat lamps placed within the isolators. This study was carried out in accordance with the Guideline for the Care and Use of Agricultural Animals in Teaching and Research (Federation of Animal Science Societies), and the protocol was approved by the Institutional Animal Care and Use (IACUC) Committee at Texas A&M University. To check the presence of *Salmonella*, a subset of chicks for each trial were humanely killed, transported and sampled upon arrival at the laboratory. Whole ceca-cecal tonsils (CCT), liver, spleen and yolk sac were aseptically removed from these neonatal chicks, incised, and cultured in 10 mL of tetrathionate enrichment broth (TEB) (Tet, Becton Dickinson, Sparks, MD) and incubated overnight at 37°C. The samples were confirmed negative for* Salmonella* by plating them onto selective BGA plates.

**Experimental design** For Trial 1, chicks in Treatment group were ora lly gavaged with 10^4^ CFU ST on day 1, and consecutively with 10^5^ CFU SE on day 2. For the control groups, two groups of chicks were orally challenged only with 10^4^ CFU ST on day 1 (ST control) or 10^5^ CFU SE on day 2 (SE control) (see Tables 1 and 2). On day 3, chickens were euthanized and cultured for recovery of *Salmonella* in CCT. To determine the incidence and frequency of SE and ST in CCT, whole CCT was enriched in TEB and incubated for 24 h at 37°C. Samples were taken from the enriched broth and subcultured on BGA plates containing 20 µg/mL NA and 25 µg/mL NO for approximately 18 h at 37°C. For the control groups, approximately 10 isolated colonies per bird were taken from each BGA and streaked as separate lines onto Mueller-Hinton plates 19. For the treatment group, approximately 20 isolated colonies per bird were streaked in the same manner onto Mueller-Hinton plates. An antibiotic disc of GM10 was placed on each streaked line of colony inoculation and the results tabulated 24 h later as either Resistant (indicating ST) or Sensitive (indicating SE). From Experiment 1, total 20 isolates were randomly selected and serogrouped using commercially available antisera to verify the accuracy of serovar identification based on sensitivity or resistance to GM10. Trial 2 was performed in the same manner as Trial 1 except that SE was used for the first challenge (10^4^ CFU) on day one, which was followed by ST challenge (10^5^ CFU) on day 2 (see Tables 1 and 2).

**Table 1:** Recovery of *Salmonella* in whole ceca-cecal tonsils (CCT) after consecutive *Salmonella* serovar challenges in day-old Lenghorn chicks in Trial 1 and Trial 2 of Experiment 1. Data is expressed as number of *Salmonella* (ST, SE or both) culture positive birds/ total number birds tested (%).

Treatment	Day	Challenge Dose (cfu)	ST recoveryCCT	SE recoveryCCT	ST and SECCT
Trial 1					
ST Control	1	10^4^	30/30 (100 %)	0/30 (0 %)	0/30 (0 %)
SE Control	2	10^5^	0/30 (0 %)	22/30 (73.30 %)	0/30 (0 %)
ST → SE	1, 2	10^4^ , 10^5^	49/60 (81.66 %)	0/60 (0 %)	11/60 (18.33 %)
					
Trial 2					
SE Control	1	10^4^	0/20 (0 %)	20/20 (100 %)	0/20 (0 %)
ST Control	2	10^5^	16/20 (80 %)	0/20 (0 %)	0/20 (0 %)
SE → ST	1, 2	10^4^ , 10^5^	0/40 (0 %)	33/40 (82.50 %)	5/40 (12.50 %)

* indicates significant difference within rows at *P* < 0.001

**Table 2:** *Salmonella* isolates recovered and serotyped in whole ceca-cecal tonsils (CCT) after consecutive *Salmonella* serovar challenges in day-old Lenghorn chicks in Trial 1 and Trial 2 of Experiment 1. Data is expressed as number of ST or SE isolates/ total number *Salmonella* isolates tested (%).

Treatment	Day	Challenge Dose (cfu)	ST recoveryCCT	SE recoveryCCT
Trial 1				
ST Control	1	10^4^	300/300 (100 %)	0/300 (0 %)
SE Control	2	10^5^	0/220 (0 %)	220/220 (100 %)
ST → SE	1, 2	10^4^ , 10^5^	1165/1200 (97.08 %)	35/1200 (2.92 %)
				
Trial 2				
SE Control	1	10^4^	0/200 (0 %)	200/200 (100 %)
ST Control	2	10^5^	160/160 (100 %)	0/160 (0 %)
SE → ST	1, 2	10^4^ , 10^5^	19/760 (2.50 %)	741/760 (97.50 %)

* indicates significant difference within rows at *P* < 0.001

### Experiment 2: Recovery of Barcode-Tagged Isogenic SE Strains After Consecutive Challenges in Day-Old Broiler Chicks

**Construction of barcode-tagged SE strains**. The method for construction of barcode-tagged strains was described previously (16). Briefly, SE 13A strain containing pKD46 that expresses the Red recombinase system was used for construction of barcode-tagged strains via electroporation. Overlapping extension PCR was used to join the three PCR products corresponding to upstream fragment (of the insertion site) plus a 6 nt random barcode, Km resistance gene and downstream fragment (of the insertion site). After electroporation, the mutants carrying the barcode sequence along with the kanamycin resistance gene inserted into a functionally neutral intergenic region between SEN1521 and SEN1522 were selected and used in this study as previously described in details (16).

**Bacterial strains and culture condition.** Two SE barcode-tagged isogenic strains (hereafter, BC1 and BC2) were incubated in Luria-Bertani (LB) broth supplemented with kanamycin (50 µg/mL) overnight at 37°C, and were harvested by centrifugation at 4°C. The cell pellet was washed three times and resuspended in distilled 0.9% saline. A suspension of 10^8^ CFU/mL was obtained by using a spectrophotometer to adjust OD_625_ = 0.147. The cell suspensions were subsequently diluted to 10^5^ CFU/mL for chick infection.

**Experimental birds.** Day-of-hatch, male broiler chicks obtained from Cobb-Vantress (Siloam Springs, AR) were placed in floor pens with a controlled age-appropriate environment. Chicks were provided *ad libitum* access to water and a balanced unmedicated corn-soybean diet meeting the nutrition requirements of poultry recommended by the NRC (1994). Adequate body temperature was maintained using heat lamps placed within the isolators. All animal handling procedures were in compliance with the Guideline for the Care and Use of Agricultural Animals in Teaching and Research (Federation of Animal Science Societies), and the experimental protocol was approved by IACUC Committee at the University of Arkansas. Twelve chickens for each trial were euthanized and sampled upon arrival at the laboratory to confirm that the chicks were *Salmonella*-negative as described in Experiment 1.

**Experimental design.** This experiment was set up to confirm if oral administration with one SE barcode-tagged strain inhibits the colonization by the other SE barcode-tagged strain in the ceca of chickens. A total of 90 day-of-hatch broiler chicks were randomly separated into six groups (*n* = 15 chicks/group). Description of the 6 treatment groups are shown in Table 3. On day three, 12 chickens from each group were euthanized. CCT and liver/spleen were collected, macerated, and suspended in 0.9% saline in 1:4 ratio in sterile bags. One mL of suspension from each bag was collected for genomic DNA isolation, and 100 µL of suspension from each bag was used for serial dilution and enumeration of CFU using BGA agar plates. Two-fold volume of tetrathionate broth (TET) was added into the remaining suspension for enrichment and detection of the positive/negative presence of *Salmonella* in each sample.

**Table 3:** Description of the treatment groups in Experiment 2*.

Treatment Groups	Challenge (Day 1 → Day 2)
1	BC1 → Saline
2	BC2 → Saline
3	Saline → BC1
4	Saline → BC2
5	BC1 → BC2
6	BC2 → BC1

BC1: SE barcode-tagged strain BC1, and BC2: SE barcode-tagged strain BC2. Saline: 0.9% sterile saline. The challenge dose was 2.5 × 10^4^ cfu per bird for both BC1 and BC2.

**Preparation of Illumina sequencing sample and analysis of DNA sequencing data. **We prepared the PCR products for quantitative profiling of barcode-tagged strains via Illumina sequencing as previously described by Yang et al. (16). Briefly, genomic DNA isolated from each of the CCT and liver/spleen samples was used to amplify the barcode regions by PCR. The PCR products were gel-purified and used as the template in the second round PCR reaction to attach Illumina-adapter sequences along with the combinatorial sample index sequences (6 nt) at both ends of the PCR products. The resulting amplicons were isolated by the ethanol purification method and were pooled together to produce a master amplicon library for MiSeq sequencing. Custom Perl scripts were used to analyze the MiSeq sequence data.

### Statistical Analysis

 The data expressed as positive/total chicks in % and the % recovery of ST or SE were compared using the chi-squared test of independence to determine the significance (*P* ≤ 0.001) (21). Barcode % recovery data within experimental groups were subjected to one way ANOVA (SAS Institute, 2002). Barcode % recoveries were expressed as means and considered significant at *P* ≤ 0.001.

## Results

### Experiment 1: Recovery of *Salmonella* After Consecutive *Salmonella* Serovar Challenges in Day-Old Lenghorn Chicks (Trials 1 and 2)

The recovery results of *Salmonella* in CCT from consecutive challenges with different *Salmonella* serovars in day-old Leghorn chicks in Trial 1 and Trial 2 are shown in Table 1. This table shows the number of *Salmonella* culture positive birds (ST positive, SE positive, or ST & SE positive) per total number birds tested. For both ST control and SE control groups in both trials, only the serovars that were used for oral administration were exclusively recovered from CCT. However, when ST was administered on day one at 10^4^ CFU, followed by the consecutive challenge of SE twenty-four hours later at 10^5^ CFU (ST→SE group in Trial 1), the birds were predominantly colonized by ST alone (81.66%; *P* < 0.001). On the contrary, in no case was SE alone recovered from any bird. Those colony isolates of SE came only from birds with mixed infections of ST and SE. In addition, when the percentage of ST or SE in all tested *Salmonella* colony isolates were determined within each group, ST and SE recovery were 97.08 and 2.92%, respectively, in the ST→SE group. On the contrary, it was 100% ST or 100% SE in the respective control groups (Table 2).

Similar results were observed in Trial 2, when chickens were challenged with SE on day one at 10^4^ CFU followed by the consecutive oral challenge of ST on day two at 10^5^ CFU (SE→ST group). In Trial 2, 82.5% of the birds were positive for SE only, the first serovar administered (Table 1). In the same SE→ST group, colonies isolated as SE from CCT were 97.5% as compared to ST colonies at 2.5% (Table 2).

### Experiment 2: Recovery of Barcode-Tagged Isogenic SE Strains After Consecutive Challenges in Day-Old Broiler Chicks

**Figure 1:**
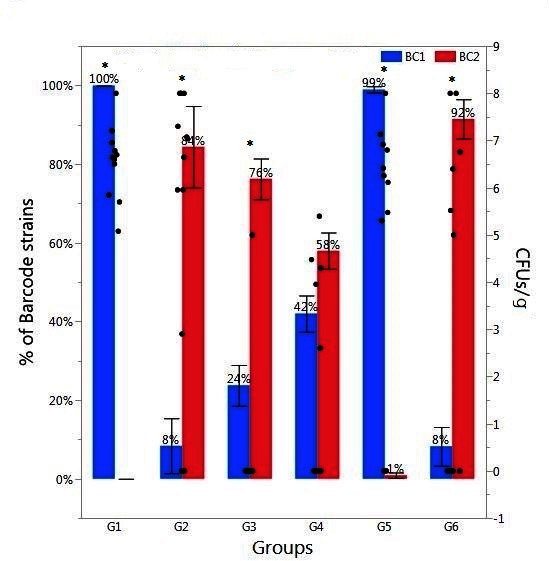
Relative abundance of *Salmonella* barcode-tagged strains (BC1 and BC2) in cecal samples in Experiment 2. The relative abundance of each barcode-tagged strain (BC1 in blue bars, and BC2 in red bars) in % (left Y axis) was determined from MiSeq data. Black dots (right Y axis) indicate CFU/g of cecal contents as determined by plating of serial dilutions. The black dots corresponding to 0 CFU/g indicate the samples in which *Salmonella* level was lower than detection limit. Day-of-hatch chickens (*n* = 12/group) were orally gavaged in Group 1 (G1) with BC1 (Day 1) and saline (Day 2); in G2 with BC2 (Day 1) and saline (Day 2); in G3 with saline (Day 1) and BC1 (Day 2); in G4, with saline (Day 1) and BC2 (Day 2), in G5 with BC1(Day 1) and BC2 (Day 2), and in G6 with BC2 (Day 1) and BC1 (Day 2). For all oral gavage with either BC1 or BC2, each chick received 2.5 × 10^4^ CFU. Asterisk (*) on the top of the bars indicates significant difference between BC1 and BC2 within the same group at *P* < 0.01.

**Figure 2:**
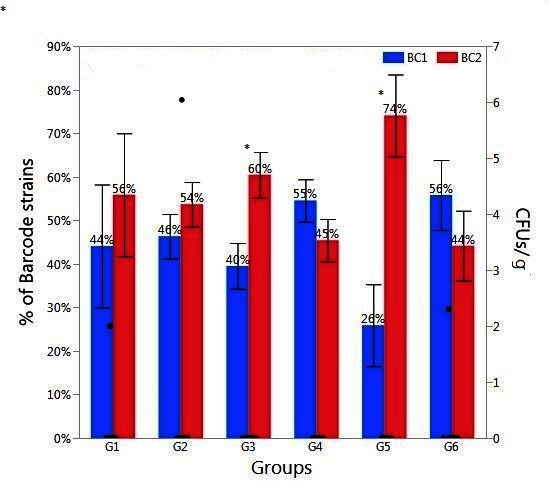
Relative abundance of *Salmonella* barcode-tagged strains (BC1 and BC2) in liver/spleen samples in Experiment 2. The relative abundance of each barcode-tagged strain (BC1 in blue bars, and BC2 in red bars) in % (left Y axis) was determined from MiSeq data. Black dots (right Y axis) indicate CFU/g of liver/spleen samples as determined by plating of serial dilutions. The black dots corresponding to 0 CFU/g indicate the samples in which *Salmonella* level was lower than detection limit. The treatment groups G1-G6 are the same to those in Figure 1. Asterisk (*) on the top of the bars indicates significant difference between BC1 and BC2 within the same group at *P* < 0.01.

The results of the percentage *Salmonella* barcode strains recovered from cecal samples enumerated from Illumina sequence data in day-old broiler chicks in Experiment 2 are summarized in Figure 1 (cecal samples) and Figure 2 (liver/spleen samples). A total of 3,138,578 sequence reads of 167 bp was obtained from the MiSeq sequencing run. The sequence reads were binned into different files according to the combinatorial index sequences corresponding to the samples from the six treatment groups (Table 3). We demanded perfect matches to the 6-nt six barcode sequences, discarding any reads without perfectly matching barcodes. The read numbers reflect only relative frequency of each barcode-tagged strain in a given sample. Therefore, the original read numbers were converted to the percentage of each barcode-tagged strain in each sample. The result from CCT indicated that oral gavage of BC1 on day one followed by saline on day two in Group 1 resulted in 100% BC1 recovery (Figure 1). Similarly, in Group 2, administration of BC2 on day one followed by saline on day two in Group 2 resulted in 84% BC2 recovery. However, oral gavage of BC1 on day one and BC2 on day two in Group 5 resulted in 99% BC1 recovery while only 1% of BC2 was recovered. The opposite effect was observed when BC2 was administered first with 92% recovery of BC2 and only 8% of BC1 recovered from CCT in Group 6. However, both BC1 and BC2 strains were isolated from ceca in the chicks from Group 3 and 4, even though only one single SE barcode-tagged strain was introduced on day two (Figure 1). The level of recovered *Salmonella* from CCT in Groups 1, 2, 5, and 6 (6.55 ± 0.22 CFU/ml^a^; 5.57 ± 0.85 ^a^; 4.87 ± 0.87 ^ab^; 3.31 ± 1.03 ^bc^, respectively) was higher than Group 3 and 4 (0.42 ± 0.42 CFU/ml^d^; 1.73 ± 0.64 ^dc^, respectively). The enrichment result from CCT exhibited a similar tendency: the percentage of *Salmonella* positive chicks from Groups 1, 2, 5, and 6 (100%, 83.33%, 75 and 50%, respectively) was higher than Group 3 and 4 (33.33% and 58.33%, respectively).

On the contrary, the results from liver/spleen samples did not reveal any apparent correlations between the infection and recovery of BCs (Figure 2). The CFU enumerations of most liver/spleen samples were below detection limit. The enrichment result showed that most of chicks were *Salmonella* negative (data now shown). Nonetheless, *Salmonella* BCs could be detected by PCR and sequencing as shown in Figure 2.

## Discussion

According to Disease Outbreak Surveillance System from 1998 to 2012, poultry was associated with 279 (25%) out of 1114 outbreaks in which the implicated source could be traced back to one food category, accounting for the highest number of outbreaks, illnesses, and hospitalizations. Among those 149 poultry-associated outbreaks were caused by a confirmed pathogen, and *Salmonella* enterica (43%) was the most common pathogens (22). Hence, understanding the mechanisms of infection of *Salmonella* in poultry is critical in order to find alternative methods to antibiotics that can eliminate or reduce this pathogen from poultry and poultry products.

Several investigators have demonstrated a significant protection from the second *Salmonella* challenge with sequential administration of *Salmonella* serovars in mice (23) or chicks (24–26). Early studies indicated that prior intravenous infection with ST could protect mice from the intravenous challenge of another *Salmonella* serovar (27, 28). Similar investigations have demonstrated that intravenous challenge of SG caused protection against subsequent intravenous challenge with SE (29). In their study, Collins et al. (29) demonstrated that SG was able to persist in the tissues therefore protecting against SE challenge. However, SP, an antigenically similar organism, was unable to establish within the tissues which apparently allowed SE to colonize. In addition, live attenuated *Salmonella* vaccines provided protection from subsequent *Salmonella* challenges within 4 weeks of vaccination (25, 30, 31). Similarly, the results of the present study confirm those by Barrow et al. (8) in which day-old chicks which received sequential *Salmonella* challenge resulted in an almost exclusive infection by the first challenge strain within 24 h. Hence, regardless of route of challenge, experimental animal or time intervals between challenges, sequential *Salmonella* challenges allow chickens to become refractory to the second *Salmonella* serovar administered. Our results also suggest that *Salmonella*-infected chicks become refractory to a second challenge serovar within 24 h, confirming previous reports of rapid induction of resistance to consecutive *Salmonella* challenge.

Several investigators have evaluated *Salmonella* transmission in commercial poultry flocks using conventional bacteriology and serological methods (32–34). These studies have helped in gaining an understanding on the impact of different phage types or housing systems on the frequency of horizontal transmission. However, comprehensive elucidation of the transmission and pathogenesis mechanisms involving interactions among multiple serovars cannot be delineated using the traditional culture methods. In the present study, two barcode-tagged SE strains were used to investigate transmission dynamics of *Salmonella* in chickens quantitatively after consecutive challenges. These strains have served as an initial conceptual proof to quantitatively track the *Salmonella* transmission routes from environment to flock, since they carry distinct barcode tags that allow them to be identified unambiguously and quantified accurately by Illumina sequencing of the barcode regions (16).

In summary, utilizing isogenic barcode-tagged strains, the population structure can be quantified to evaluate the patterns of SE infection and dissemination in chickens, and determine whether infection of neonatal chicks with one *Salmonella* strain excludes the infection by a second strain. Our Illumina sequence data indicated that any BC strain used for infection on day one became the predominant strain, whether there was a second infection by the other BC strain (Group 5 and 6 in Figure 1) or not (Group 1 and 2 in Figure 1). Unexpectedly, there was a mixed population of two BC strains when the first infection by BC strain was delayed to day 2 (Group 3 and 4 in Figure 1). In case of Group 3 and 4, the chicks were infected only by one BC strain (BC1 and BC2, respectively) on day 2, but both BC strains could be recovered at significant levels. It is important to note that the level of recovered *Salmonella* from CCT was significantly lower in Group 3 (0.42 ± 0.42 CFU/ml) and 4 (1.73 ± 0.64 CFU/ml) as compared to other groups (≥3.31 ± 1.03). The result indicates the possible aerosol transmission may exist from other groups in the same isolation room through the respiratory tract (35).

Based on the dilution, counting and enrichment method, the *Salmonella* level was too low to be detected for most liver/spleen samples (data not shown). However, both *Salmonella* BC strains could be detected in most of the liver/spleen samples by using PCR and sequencing (Figure 2). This result indicated that this strategy is more sensitive than the traditional culture method. Surprisingly, all groups had significant levels of both BC1 (≥26%) and BC2 (≥44%) (Figure 2), whether it was infected by one or two BC strains. This also provides additional evidence for the aerosol transmission through the respiratory tract and it indicates that dissemination into internal organs (liver and spleen) might be more efficient than dissemination into ceca after infection through the respiratory tract. There is a possibility that once *Salmonella* infection appears in the tracheal route, they may migrate into different body sites more rapidly with higher efficiency than infection through the oral route. *Salmonella* can replicate in the respiratory macrophages and transport from the lungs to the secondary lymphoid organs, such as spleen, and spread systemically to liver and potentially to cecal tonsil later (36, 37).

Alternative explanations for the result presented in Figure 2 include inaccurate differentiation between BC1 and BC2 or instability of barcode-tags. However, we used 2-step PCR to amplify barcode regions using 2 sets of primers, enhancing specificity of amplification and ensuring barcode tags recovered are from the originally inserted chromosomal locus. When the sequence reads were processed, we demanded perfect matches to 6nt barcode sequences, discarding any reads with no perfect matches. For these reasons, we argue that the observed barcode profiles accurately reflect relative abundance of BC1 and BC2 in the given samples. It is also important to note that we cannot exclude the possibility that the barcode sequences could have been amplified from dead cells or DNA, based on the fact that most liver/spleen samples were *Salmonella*-negative on enrichment.

In order to better comprehend the implications of CE and intratracheal infection of *Salmonella* in commercial poultry, larger scale experiments are necessary to assess additional environmental and host factors. Nevertheless, the current experiment further confirmed that the use of barcode-tagged strains is an original and an effective method to understand the dynamics of *Salmonella* infection, which provides valuable opportunities to develop and improve effective measures to control *Salmonella* in poultry flocks. Currently, studies to evaluate and confirm our previous work (35) that demonstrated the importance of airborne transmission of *Salmonella* via an intratracheal route versus oral infection are conducted using these SE barcode-tagged strains.

## Ethics Statement

All animal handling procedures were in compliance with Institutional Animal Care and Use Committees at Texas A & M University, and University of Arkansas.

## Author Contributions

GT, BH, and YK designed the experiments. YY and PR conducted experiments and analyzed the data. YY, GT, JL, XH, and SR wrote the manuscript. YY and YMK revised the manuscript.

## Conflict of Interest Statement

The authors declare that the research was conducted in the absence of any commercial or financial relationships that could be construed as a potential conflict of interest.
